# Dual Independent Roles of the p24 Complex in Selectivity of Secretory Cargo Export from the Endoplasmic Reticulum

**DOI:** 10.3390/cells9051295

**Published:** 2020-05-22

**Authors:** Sergio Lopez, Ana Maria Perez-Linero, Javier Manzano-Lopez, Susana Sabido-Bozo, Alejandro Cortes-Gomez, Sofia Rodriguez-Gallardo, Auxiliadora Aguilera-Romero, Veit Goder, Manuel Muñiz

**Affiliations:** 1 Department of Cell Biology, University of Seville, 41012 Seville, Spain; serglom@us.es (S.L.); anamaripl@yahoo.es (A.M.P.-L.); jmanzano@us.es (J.M.-L.); ssabido@us.es (S.S.-B.); acgomez@us.es (A.C.-G.); srodriguez13@us.es (S.R.-G.); auxi@us.es (A.A.-R.); 2 Instituto de Biomedicina de Sevilla (IBiS), Hospital Universitario Virgen del Rocío/CSIC/Universidad de Sevilla, 41012 Seville, Spain; 3 Department of Genetics, University of Seville, 41012 Seville, Spain; vgoder@us.es

**Keywords:** endoplasmic reticulum, cargo receptor, p24 complex, secretory cargo, bulk flow

## Abstract

The cellular mechanisms that ensure the selectivity and fidelity of secretory cargo protein transport from the endoplasmic reticulum (ER) to the Golgi are still not well understood. The p24 protein complex acts as a specific cargo receptor for GPI-anchored proteins by facilitating their ER exit through a specialized export pathway in yeast. In parallel, the p24 complex can also exit the ER using the general pathway that exports the rest of secretory proteins with their respective cargo receptors. Here, we show biochemically that the p24 complex associates at the ER with other cargo receptors in a COPII-dependent manner, forming high-molecular weight multireceptor complexes. Furthermore, live cell imaging analysis reveals that the p24 complex is required to retain in the ER secretory cargos when their specific receptors are absent. This requirement does not involve neither the unfolded protein response nor the retrograde transport from the Golgi. Our results suggest that, in addition to its role as a cargo receptor in the specialized GPI-anchored protein pathway, the p24 complex also plays an independent role in secretory cargo selectivity during its exit through the general ER export pathway, preventing the non-selective bulk flow of native secretory cargos. This mechanism would ensure receptor-regulated cargo transport, providing an additional layer of regulation of secretory cargo selectivity during ER export.

## 1. Introduction

Nearly a third of all eukaryotic proteins are synthesized in the endoplasmic reticulum (ER) and subsequently transported by the secretory pathway through the Golgi complex to their functional destination, either the cell surface or the endo-lysosomal system [1,2]. Newly synthesized proteins are translocated into the ER where they become folded, assembled and post-translationally modified by glycosylation and disulfide bond formation. Once correctly matured, secretory proteins are then separated from incompletely folded proteins and ER residents to be incorporated as cargo into COPII-coated transport vesicles that transfer them forward to the Golgi apparatus. COPII vesicles are generated by the sequential assembly of the cytosolic COPII coat components, the small GTPase Sar1 and the two protein dimers Sec23/Sec24 and Sec13/Sec31, which locally bend the ER membrane at specific domains called ER exit sites (ERES) [3]. Secretory proteins can be selectively captured into nascent COPII vesicles by transmembrane receptors or adaptors that physically link each specific cargo with the COPII coat [2,4]. In contrast to this selective and active cargo capture, secretory proteins have been shown also to enter COPII vesicles by non-selective and passive bulk flow. However, to what extent ER export is driven by selective cargo capture versus non-selective bulk flow is still unknown [5].

The p24 complex is a heteromeric protein complex formed by members of the conserved p24 family protein, which are abundant type I transmembrane proteins that cycle between ER and Golgi apparatus thanks to cytosolic COPII and COPI coat-binding signals [6,7,8,9,10,11]. The p24 complex has been shown to play several functions during its bidirectional transport between the ER and Golgi at the early secretory pathway. First, the p24 complex facilitates the formation of COPI retrograde transport vesicles from the *cis*-Golgi [12,13]. Second, the p24 complex functions as a specialized cargo receptor by promoting the efficient ER export of a class of lipid-anchored cell surface proteins, glycosylphosphatidylinositol (GPI)-anchored proteins (GPI-APs) [14,15,16,17,18,19,20,21]. Finally, although facilitating GPI-AP export, the p24 complex contributes to the ER retention of resident proteins and some misfolded non-GPI-APs [22,23].

The special structure and composition of the GPI anchor lead GPI-APs to be differentially trafficked along the secretory pathway [24,25,26]. In yeast, GPI-APs are segregated from other secretory proteins in the ER, and subsequently incorporated into distinct COPII vesicles [27,28]. This implies the existence of at least two different and parallel ER exit pathways, one of which exports GPI-APs and the other, the rest of the secretory proteins [24,25]. According to its role as a cargo receptor, the p24 complex travels together with its cargo, the GPI-APs, into the same specialized COPII vesicles. However, unexpectedly, the p24 complex was also found in the COPII vesicles that carry non-GPI-anchored cargos [16]. The fact that a subpopulation of the p24 complex exits the ER via the non-GPI-AP pathway may suggest additional functions to its role as a specific cargo receptor. To gain more insights into this issue, we have identified binding partners of the p24 complex member Emp24 by performing a pull-down assay from the ER membrane fraction. We found that the p24 complex interacts with several other cargo receptors that operate in the non-GPI-AP or general ER export pathway forming High-Molecular Weight multireceptor complexes at the level of the ER exit sites. Moreover, we show that the ER retention of non-GPI-anchored cargos caused by the absence of their specific receptors requires the presence of the p24 complex, suggesting its role in preventing the non-selective bulk flow of native secretory cargo in the general ER export pathway.

## 2. Materials and Methods

### 2.1. Media and Growth Conditions

Yeast cultures were grown at 24 °C in rich YP medium (1% yeast extract, 2% peptone) supplemented with 0.2% adenine and containing 2% glucose (YPD) as the carbon source or in synthetic minimal medium (0.15% yeast nitrogen base, 0.5% ammonium sulfate) supplemented with the appropriate amino acids and bases as nutritional requirements, and containing 2% glucose (SD) as the carbon source.

### 2.2. Yeast Strains and Plasmids

Strains of *Saccharomyces cerevisiae* and plasmids used for this work are listed in Table 1 and Table 2, respectively.

### 2.3. Proteomic Identification of Binding Partners for Emp24-TAP

Emp24-TAP interactors were proteomically identified from an enriched ER membrane fraction as described [19]. In brief, 15 g of yeast cells expressing Emp24-TAP were lysed by grinding in liquid nitrogen, after which, cell debris was removed by centrifugation. The supernatant was then centrifuged at 13,000× *g* for 15 min at 4 °C. The enriched ER membrane pellet was resuspended in buffer containing 1% digitonin (AppliChem). The extract was incubated for 3 h with IgG-coupled magnetic beads (Dynal). After washing and elution, the eluted proteins were analyzed by SDS-PAGE, and different gel regions were excised and prepared for in-gel digestion with trypsin. Peptide samples were subjected to liquid chromatography tandem mass spectrometry (LC-MS/MS) using a LCQ Deca XP Plus ion trap mass spectrometer (Thermo Electron), [29,30]. Proteins identified by at least two tryptic peptides or more were characterized using Swiss-Prot/TrEMBL/UniProt database (UniProt Consortium, 2019) [31] and YeastMine (http://www.yeastgenome.org).

### 2.4. Native Co-Immunoprecipitation

Experiments of native co-immunoprecipitation of Erv14-mCi for subsequent immunoblotting analysis were performed on enriched ER fractions as described [15]. In brief, 100 optical density 600 (OD600) units of yeast cells expressing Erv14-mCi were washed twice with TNE buffer (50 mM Tris-HCl (pH 7.5), 150 mM NaCl, 5 mM EDTA, 1 mM phenylmethylsulfonylfluoride, and protease inhibitor cocktail; Roche Diagnostics) and disrupted with glass beads, after which, cell debris and glass beads were removed by centrifugation. The supernatant was then centrifuged at 13,000× *g* for 15 min at 4 °C. The enriched ER membrane pellet was resuspended in TNE buffer, and digitonin was added to a final concentration of 1%. The suspension was incubated for 1 h at 4 °C with rotation, after which insoluble components were removed by centrifugation at 17,000× *g* for 60 min at 4 °C. For immunoprecipitation of Erv14-mCi, the sample was first preincubated with bab agarose beads (ChromoTek) at 4 °C for 1 h and subsequently incubated with GFP-Trap_A (ChromoTek) at 4 °C for 3 h. The immunoprecipitated beads were washed five times with TNE buffer containing 0.2% digitonin, eluted with SDS sample buffer, resolved on SDS-PAGE, and analyzed by immunoblot.

### 2.5. Separation of High-Molecular-Weight Protein Complexes by Density Gradient Centrifugation

100 OD600 units of log-phase cells were disrupted in TNE buffer with glass beads, after which, cell debris and glass beads were discarded by centrifugation. The resultant supernatant was centrifuged at 13,000× *g* for 15 min at 4 °C. The pellet containing the enriched ER membrane fraction was solubilized in TNE buffer with 1% digitonin for 1 h at 4 °C with rotation. The extract was cleared by centrifugation (17,000× *g* for 1 h at 4 °C) and loaded on top of a 5%–30% sucrose step gradient in TNE buffer with 0.2% digitonin. The step size was 5% sucrose. Centrifugation was performed for 4 h at 50,000 rpm and 4 °C in a TLS-55 rotor (Beckman Coulter). The gradient was fractionated from top to bottom, and fractions were subjected to native co-immunoprecipitation or processed for SDS-PAGE and immunoblotting.

### 2.6. Fluorescence Live Cell Microscopy

For fluorescence live cell microscopy of Mid2-Venus, Gnt1-GFP, CPY-GFP and Gap1-ub-GFP, log-phase cells grown in minimal media were observed directly. For fluorescence live cell microscopy of Cwp2-Venus and Gas1-GFP, log-phase cells were also grown in minimal media and incubated for 2 h in rich media (YPD). Then, these cells were collected by centrifugation, washed twice with PBS, and incubated at least 15 min on ice before being examined under the microscope as described previously [32]. Images were captured using a Leica DMi8 microscope equipped with an objective lens (HCX PL APO 1003/1.40OIL PH3 CS), L5 (GFP) filter, a Hamamatsu camera, and Application Suite X (LAS X) software, as described previously [15].

### 2.7. Staining of Bud Scars with Calcofluor White

Log-phase yeast cells grown in minimal media were collected by centrifugation, resuspended in calcofluor white stain at a concentration of 0.1 mg/mL [14] and incubated for 10 min at room temperature. After calcofluor white treatment, cells were washed three times in distilled water and observed directly under a Leica DMi8 fluorescence microscope. Haploid cells having six or more bud scars were examined for an axial or nonaxial budding pattern.

### 2.8. Statistical Analysis

All values in the figures are expressed as the mean ± SD. Data were evaluated with GraphPad Prism Version 5.01 software. Statistical significances were assessed using the analysis of the variance (ANOVA) with Tukey’s post hoc test for multiple comparisons. The level of significance was set at 0.05.

## 3. Results

### 3.1. The p24 Complex Associates with Other ER Cargo Receptors into High Molecular Complexes in a COPII-Dependent Manner

**Figure 1:**
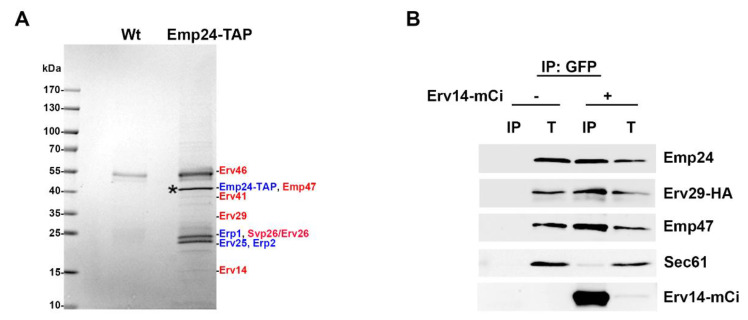
The p24 complex interacts with other cargo receptors at the ER membrane. (**A**) Coomassie staining of the p24 protein Emp24-TAP interactors. Cargo receptors (red) and p24 proteins (blue) that interact with Emp24-TAP (blue) are indicated. Enriched ER membrane fractions of wild-type yeast cells or cells expressing Emp24-TAP were isolated and solubilized with 1% digitonin. The extract was incubated with IgG-coupled magnetic beads and co-precipitated proteins were prepared and resolved by SDS-PAGE. Visualized bands and regions from the control reaction were subjected to in-gel digestion with trypsin and were analyzed by LC-MS/MS techniques (Figure S1, Tables S1 and S2). The star indicates the Emp24-TAP. (**B**) Co-immunoprecipitation assay between Erv14-mCi and other cargo receptors. Enriched ER membrane fractions of wild-type cells expressing Erv29-HA with or without expressing Erv14-mCi were solubilized, and immunoprecipitated (IP) with anti-GFP antibody, followed by immunoblotting with anti-Emp24, anti-HA, anti-Emp47, anti-Sec61 and anti-GFP antibodies. T represents a 0.5% of the solubilized input material.

The yeast p24 protein complex acts as a specific cargo receptor by promoting the efficient transport of GPI-APs from the ER to the Golgi through their specialized COPII-dependent pathway [25]. However, the p24 complex was also found in the COPII vesicles that export from the ER the rest of secretory proteins together with their respective cargo receptors [16]. This dual routing from the ER to the Golgi suggests a possible additional role for the p24 complex in the general ER export pathway. To address this possibility, we first identified ER binding partners of the p24 complex member Emp24 by performing a native co-immunoprecipitation assay. Emp24 was chromosomally tagged with the TAP tag and affinity purified with IgG-coupled magnetic beads from detergent-solubilized ER membrane fractions. Co-precipitated proteins were next identified by tandem mass spectrometry (Figure 1 and Figure S1, Tables S1 and S2). As a specificity control, we used a wild-type strain without tagged protein. Validity of the pull-down assay was confirmed by the presence of the other p24 complex members Erv25, Erp1 and Erp2 among the binding partners of Emp24-TAP [6,33]. Interestingly, we also found in the eluate other cargo receptors that operate in the non-GPI-AP or general ER export pathway, including Erv14, Erv29, Svp26/Erv26 or Emp47. These cargo receptors are largely heterogeneous in terms of structure and cargo recognition motifs. Erv14 mediates the ER export of plasma membrane proteins with long transmembrane domains, Erv29 sorts soluble secretory proteins into COPII vesicles, Erv26 promotes the efficient ER exit of Golgi mannosyltransferases and Emp47 is required for the efficient ER export of some glycoproteins [2,5,34,35,36,37]. In addition, Erv41 and Erv46 were also found in the eluate as p24 interactors. They form a protein complex that has been reported to function as a cargo receptor in both anterograde and retrograde transport pathways [38,39]. To confirm the proteomic results, yeast cells expressing the cargo receptor Erv14 tagged with mCitrine (mCi) were analyzed by native co-immunoprecipitation followed by conventional immunoblotting. As seen in Figure 1B, immunoprecipitation in detergent-solubilized ER membrane fractions revealed that the Erv14-mCi co-precipitated with Emp24, Erv29-HA and Emp47. These physical interactions were specific for cargo receptors because the unrelated translocon subunit Sec61 was not recovered after Erv14-mCi immunoprecipitation. Furthermore, Erv14-mCi also co-precipitated with the core p24 complex member Erv25, indicating that the p24 proteins bind other cargo receptors as a complex (Figure S2).

**Figure 2:**
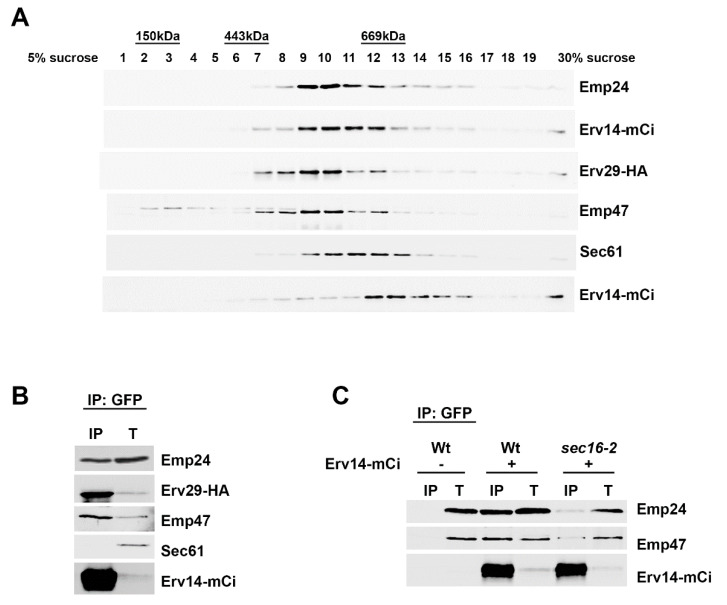
ER cargo receptors associate into high molecular complexes in a COPII-dependent manner. (**A**) Isolation of high weight molecular complexes. Enriched ER membrane fractions of wild-type cells expressing Erv14-mCi and Erv29-HA were solubilized with 1% digitonin and were fractionated by 5%–30% sucrose density gradient centrifugation. Fractions were collected from each gradient and directly analyzed by SDS-PAGE followed by immunoblotting with different antibodies. The asterisk represents a non-specific protein crossreacting with the anti-Emp47 antibody. (**B**) Fractions 9 and 10 were mixed and immunoprecipitated (IP) with anti-GFP antibody, followed by immunoblotting with anti-Emp24, anti-HA, anti-Emp47, anti-Sec61 and anti-GFP antibodies. The co-IP efficiency for Emp24, Erv29 and Emp47 was 0.8%, 4.1% and 2.5% respectively. T represents a 0.5% of the solubilized input material. (**C**) Physical interactions among ER cargo receptors depends on the COPII regulator Sec16. Enriched ER membrane fractions of wild-type and *sec16-2* mutant cells expressing Erv14-mCi, which were previously incubated at 37 °C, were solubilized, and immunoprecipitated (IP) with anti-GFP antibody, followed by immunoblotting with anti-Emp24 and anti-Emp47. T represents a 0.5% of the solubilized input material.

Next, we determined whether these cargo receptors that interact with each other are present in larger protein complexes. For this purpose, ER membrane fractions from yeast cells expressing Erv14-mCi and Erv29-HA were solubilized with 1% digitonin and subjected to sucrose density gradient centrifugation. The resultant gradient fractions were analyzed by SDS-PAGE and immunoblotting with specific antibodies against mCi, HA, Emp24 and Emp47. As observed in Figure 2A, Emp24, Erv14-mCi, Erv29-HA and Emp47 cargo receptors were found in high density fractions. Their sedimentation patterns showed certain heterogeneity, suggesting the formation of heterogeneous receptor complexes of distinct composition and size (higher than 450 kDa). Nevertheless, all four of the receptors peaked around fraction 10. To verify that they were indeed associated with high molecular weight complexes, Erv14-mCi immunoprecipitation in detergent-solubilized fractions 9 and 10 was performed. The subsequent immunoblot analysis of the eluate revealed that Emp24, Erv29 and Emp47 specifically co-precipitated with Erv14-mCi, although Emp24 was less efficiently recovered (Figure 2B). This result may indicate that only a subpopulation of the p24 complex travels together with the other cargo receptors through the general ER export pathway.

Cargo receptors work by linking their cargos with the COPII coat and they exit the ER as major components of COPII vesicles [5]. To determine whether the formation of the multireceptor complexes requires the COPII machinery, we analyzed the cargo receptor interactions in vivo by native co-immunoprecipitation in the COPII thermosensitive mutant strain *sec16-2*. Sec16 is a COPII accessory protein peripherally associated with the ER membrane that regulates the GTP cycle of the COPII coat for vesicle biogenesis at ER exit sites [40,41,42,43]. As shown in Figure 2C, in *sec16-2* mutant cells, the co-immunoprecipitation of Erv14-mCi with Emp24 and Emp47 is greatly reduced at restrictive temperature (37 °C). This result suggests that the multireceptor complexes are assembled at the level of ER exit sites during the COPII vesicle formation.

### 3.2. The p24 Complex Prevents the Non-Selective ER Exit of Secretory Cargos in the Absence of Their Specific Receptors

**Figure 3:**
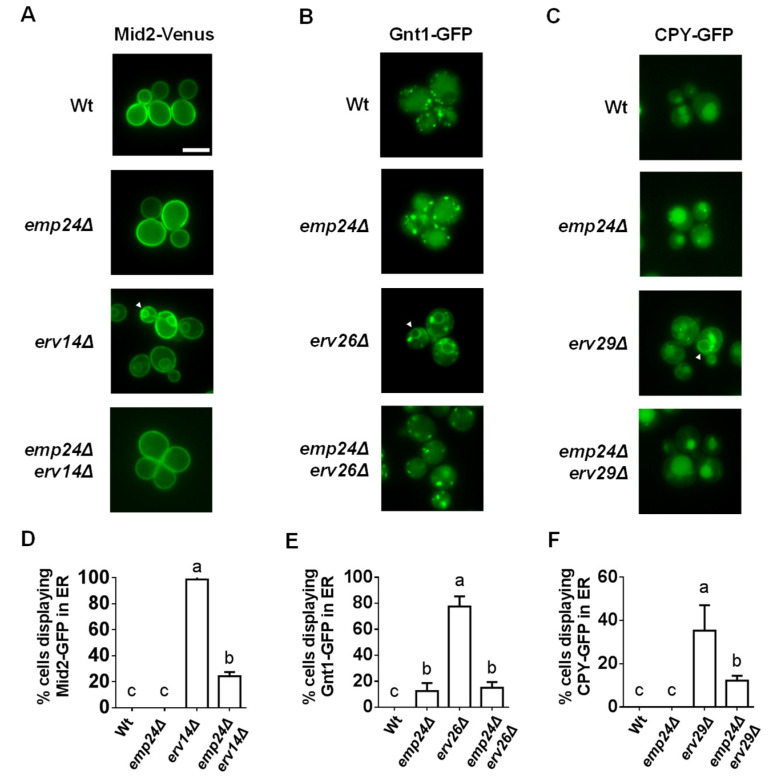
The p24 complex is required for the ER retention of different secretory cargos in the absence of their receptors. (**A**–**C**) Fluorescent live images of wild-type and different deletion strains expressing the secretory cargos Mid2-GFP (**A**), Gnt1-GFP (**B**) or CPY-GFP (**C**). The white arrow points out the perinuclear ER. Scale bar represents 5 µm. (**D**–**F**) Quantification of several micrographs described in (**A**,**B**) or (**C**). The graphs plot the average percentage of cells displaying perinuclear ER pattern. n, number of cells plotted ≥ 100. Different letters denote significant differences between strains (one-way ANOVA test, *p* ≤ 0.05).

Emp24 is always assembled with the other p24 proteins into the p24 complex. Thus, the fact that Emp24 associates with other cargo receptors in large multiprotein complexes opens the possibility that the p24 complex plays in the general ER export pathway a different role as the specific cargo receptor role played in the GPI-AP pathway. Because previous studies have shown that the p24 complex participates in the ER retention of resident proteins such as chaperones and some misfolded non-GPI-APs [22,23], we tested by live fluorescence microscopy whether the p24 complex contributes to prevent the bulk flow or non-selective exit of secretory cargos from the ER. For this purpose, we used the deletion of *EMP24* that destabilizes the other proteins of the p24 complex, leading to a complete loss of p24 complex function [6]. Figure 3A addresses the effect of the *EMP24* deletion in the ER retention of diverse secretory cargos by the absence of their specific receptors. Mid2-GFP, a plasma membrane-localized protein, was accumulated in the ER in the absence of its cargo receptor Erv14, displaying the ER-characteristic nuclear ring staining [34]. In contrast, when Mid2-GFP was expressed in *emp24*Δ *erv14*Δ double-mutant cells, no ER staining was observed and Mid2-GFP was mainly localized at the cell surface. The same release effect was observed for other secretory cargos. The N-acetylglucosaminyltransferase Gnt1-GFP localizes at the Golgi apparatus, displaying the typical Golgi-dotted pattern for yeast cells [44]. As expected, Gnt1-GFP was partially retained at the ER when its cargo receptor, Erv26, was deleted. However, in the *emp24*Δ *erv26*Δ double-mutant cells, Gnt1-GFP was mostly detected in the Golgi. Additionally, the vacuolar carboxypeptidase Y enzyme CPY-GFP, was partially accumulated in the ER in the absence of its cargo receptor Erv29, but this ER retention was also reduced in the *emp24*Δ *erv29*Δ double-mutant cells.

**Figure 4:**
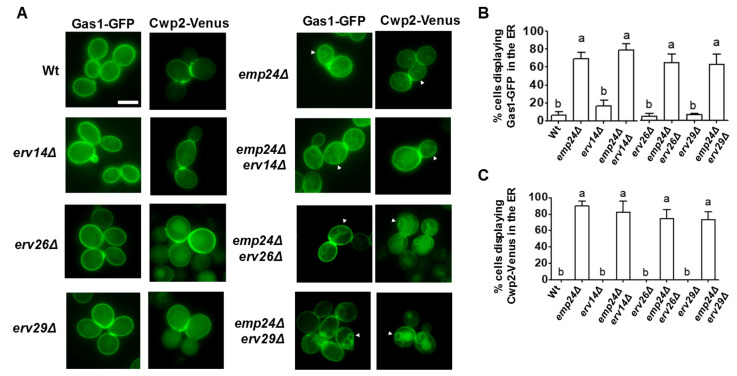
The p24 complex is still required for the ER export of GPI-APs in the absence of other cargo receptors. (**A**) Fluorescent live images of wild-type and different deletion strains expressing Gas1-GFP or Cwp2-Venus. The white arrow points out the perinuclear ER. Scale bar represents 5 µm. (**B**,**C**) Quantification of several micrographs described in (**A**). The graphs plot the average percentage of cells displaying perinuclear ER pattern. n ≥ 100. Different letters denote significant differences between strains (one-way ANOVA test, *p* ≤ 0.05).

Thus, these results indicate that ER retention of a specific cargo in the absence of its selective receptor is compromised by the p24 loss of function. However, this lack of ER retention was not observed for their own specific cargos of the p24 complex, the GPI-APs. As shown in Figure 4, two different GPI-APs, Gas1-GFP and Cwp2-Venus, localize at the cell surface in wild-type and *erv14*Δ, *erv26*Δ, *erv29*Δ receptor mutant cells while they are detected in the ER in the *emp24*Δ and *emp24*Δ *erv14*Δ, *emp24*Δ *erv26*Δ or *emp24*Δ *erv29*Δ mutant cells. Therefore, all together, these results strongly suggest that one of the p24 complex functions during the ER export is to prevent the non-selective cargo export from the ER and that this function is independent of its role as a cargo receptor for GPI-APs.

**Figure 5:**
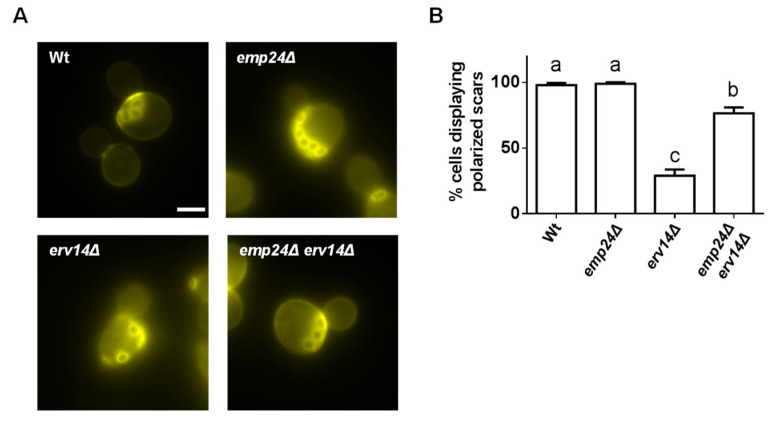
A secretory cargo retained at the ER by the p24 complex in the absence of its receptor is functional. (**A**) Budding patterns in wild-type (WT) and *erv14*Δ strains. Yeast cells were stained with calcofluor to visualize bud scars. Scale bar represents 5 µm. Wild-type and *emp24*Δ cells display the typical axial budding pattern whereas *erv14*Δ cells exhibit nonaxial budding patterns. However, the axial budding pattern is recovered in the *emp24*Δ*erv14*Δ double mutant cells. (**B**) Quantification of several micrographs described in (**A**). The graph plots the average percentage of cells displaying polarized scars. n ≥ 100. Different letters denote significant differences between strains (one-way ANOVA test, *p* ≤ 0.05).

To get more insight into the cargo-receptor independent role of the p24 complex in selectively and fidelity of cargo export from the ER, we tested whether those secretory cargos retained at the ER by the p24 complex in the absence of their respective receptors can reach a functional native status. For this reason, we analyzed the functionality of a specific cargo of the Erv14 receptor, the plasma membrane protein Axl2, in the *emp24*Δ *erv14*Δ double mutant strain. Haploid yeast requires Axl2 to bud in an axial budding pattern, where the new bud site occurs adjacent to a structure called the bud scar, leftover from the previous cell division [45]. Therefore, the axial budding pattern results in a continuous chain of bud scars on older mother cells that can be visualized by staining with the fluorescent dye calcofluor (Figure 5). In contrast to wild-type cells, the *erv14*Δ mutant cells display a non-axial pattern, since Axl2 is retained at the ER and thus, cannot reach the plasma membrane [46]. However, the axial pattern is substantially recovered in *emp24*Δ *erv14*Δ double mutant cells, indicating that this secretory cargo retained in the ER by the p24 complex is mostly functional.

### 3.3. Secretory Cargo ER Retention by the p24 Complex Involves Neither the UPR nor the Retrograde Transport from the ER

**Figure 6:**
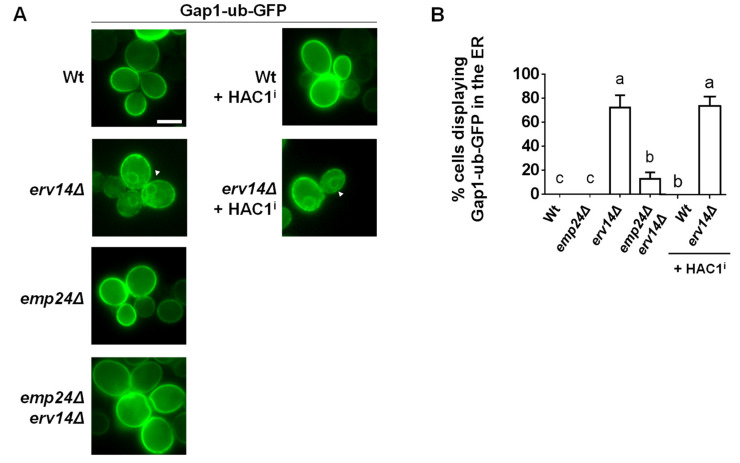
The chronic activation of the UPR does not cause the ER escape of a secretory cargo from the ER in the absence of its corresponding receptor. (**A**) Fluorescent live images of wild-type and different deletion strains expressing the Erv14 cargo GAP1-GFP mutated at ubiquitination sites (Gap1-ub-GFP) and without or with the plasmid pDN390 expressing spliced HAC1 mRNA (HAC1^i^) to chronically activate the UPR. The white arrow points out the perinuclear ER. Scale bar represents 5 µm. (**B**) Quantification of several micrographs described in (**A**). The graph plots the average percentage of cells displaying perinuclear ER pattern n ≥ 100. Different letters denote significant differences between strains (one-way ANOVA test, *p* ≤ 0.05).

Deletion of p24 genes in yeast has been previously shown to cause the ER escape of the resident chaperone Kar2 and its subsequent secretion to the extracellular media [23]. However, this phenotype has been attributed to the fact that the p24 deletion mutant strains have chronically activated the unfolded protein response (UPR), which increases Kar2 expression [47]. Therefore, it is possible that a chronic UPR triggered by *EMP24* deletion leads also to the ER escape of secretory cargos in the absence of their receptors. To address this possibility, we transformed different strains with a plasmid expressing spliced *HAC1* mRNA [48]. *HAC1* is a transcriptional activator of UPR response genes whose functional expression is driven by non-conventional cytosolic splicing of its mRNA. Thus, expression of spliced *HAC1* causes a constitutively active UPR. Under these conditions, we observed that GAP1-ub-GFP, a plasma membrane protein and specific cargo for Erv14 receptor [28], was still retained at the ER in the erv14Δ cells and localized at the plasma membrane in wild-type cells (Figure 6). These results show that the activated UPR does not cause the ER escape of secretory cargo, and therefore, the function of the p24 complex on ER retention is independent of UPR activation. This is in agreement with previous observations that ER leakage of soluble chaperones in the absence of the p24 function occurred independently of the UPR [22,49].

**Figure 7:**
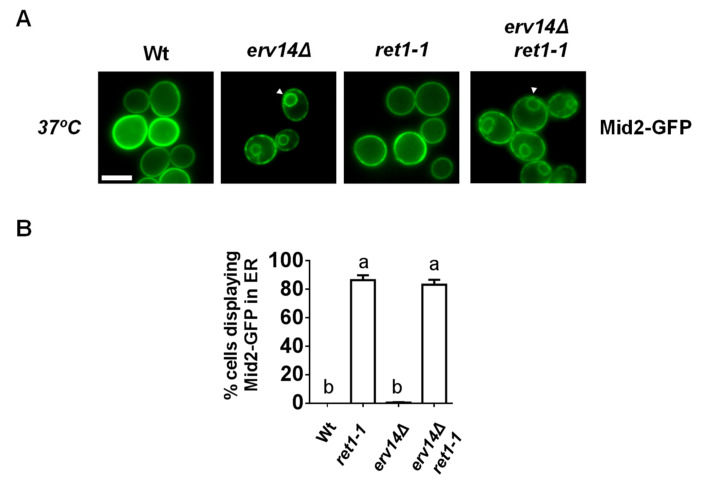
An active COPI-dependent retrograde transport is not required for ER retention of a cargo expressed in the absence of its specific receptor. (**A**) Live images of wild-type, *erv14*Δ, *ret1-1* and *erv14*Δ *ret1-1* expressing Mid2-GFP at 37°C. Scale bar, 5 μm. (**B**) Quantification of several micrographs described in (**A**). The graph plots the average percentage of cells displaying Mid2-GFP in the ER. n ≥ 100. Different letters denote significant differences between strains (one-way ANOVA test, *p* ≤ 0.05).

The p24 complex is continuously cycling between the ER and Golgi and it has been also shown to play a role in the COPI coat-dependent retrograde transport from the Golgi to the ER by facilitating the formation of COPI-coated vesicles at the Golgi membrane [12]. This retrograde transport pathway is involved in the recycling of ER escaped misfolded proteins and resident proteins, thereby contributing to the ER retention and export selectivity [5]. Therefore, it is possible that the *EMP24* deletion impairs the retrieval from the Golgi of those cargos escaped from the ER due to the absence of their specific receptors. We examined the influence of a mutation that generally perturbs COPI function, such as the temperature-sensitive *ret1-1* mutation in α-COP subunit [50], on the ER retention of the Erv14 cargo Mid2-GFP in the *erv14*Δ cells. Although the *ret1-1* mutation is defective for retrograde transport at restrictive temperature, 37 °C (Figure S3), Mid2-GFP still displayed an ER signal in the double mutant *ret1-1 erv14*Δ (Figure 7). These results indicate that the function of the p24 complex in the COPI-mediated recycling from the Golgi to the ER is not involved in the ER retention of cargos expressed in the absence of their receptors. Instead, the p24 complex must prevent the non-selective bulk flow of secretory cargo during its own export from the ER. This is consistent with previous observation that ER leakage of a soluble chaperone in the absence of the p24 function was observed in vitro using microsomes [14].

## 4. Discussion

ER export of newly synthesized secretory proteins is an essential and highly selective process, although the molecular bases of transport fidelity and selectivity are still not well understood. In yeast, at least two parallel ER export pathways have been described to deliver selected cargo proteins to the Golgi [25]. One pathway specifically transports GPI-APs, whereas the other pathway transports the rest of the secretory proteins. The GPI-AP pathway depends on the p24 complex, which acts as a specific cargo receptor by linking the cytosolic COPII coat with GPI-APs to thereby incorporate them into specialized COPII vesicles. The p24 complex can as well exit the ER in the same COPII vesicles that export non-GPI-APs with their specific cargo receptors [16]. In this study, we have biochemically identified several of these cargo receptors for non-GPI-APs as interactors of the p24 complex. In particular, we found that the p24 protein Emp24 specifically interacts with Erv14, Erv26, Erv29 and Emp47, which facilitate the ER export of a large diversity of secretory cargos including plasma membrane proteins with long transmembrane domains (Erv14), Golgi mannosyltransferases (Erv26), soluble secretory proteins (Erv29) or specific glycoproteins (Emp47) [5]. In addition, the Erv41/46 protein complex, which has been reported to act as both an anterograde and retrograde cargo receptor during the bidirectional transport between the ER and the Golgi, was also found to interact with Emp24 [38,39]. In line with these findings, a previous study also identified SURF4 and ERGIC-53, the mammalian orthologues of Erv29 and Emp47 respectively, as interactors of the mammalian p24 proteins [51]. Furthermore, by using sucrose density gradient centrifugation of these digitonin-solubilized ER membrane fractions, we revealed that cargo receptors associate into high molecular weight multiprotein complexes at the ER membrane. Interestingly, the formation of these multireceptor complexes must occur during the biogenesis of the COPII vesicle since it was found to depend on the COPII accessory protein Sec16. Sec16 is a peripheral ER protein that cooperates with the major COPII cargo adaptor Sec24 to modulate the GTP cycle of the COPII coat. It has been proposed that cargo binding to Sec24 would engage Sec16 to inhibit Sar1 GTPase activity, and thereby, delay COPII vesicle release until cargo loading is completed, avoiding then the premature formation of empty carriers [43,52,53]. Consistent with this possible mechanism, oligomerization of a cargo receptor has been shown to direct protein sorting into COPII vesicles [54]. Therefore, the assembly of large multireceptor complexes resulting in a high degree of oligomerization could synergistically increase the delay of vesicle scission, and thus, enhance the efficiency and selectivity of sorting for each cargo receptor and, consequently, for their specific cargos.

**Figure 8:**
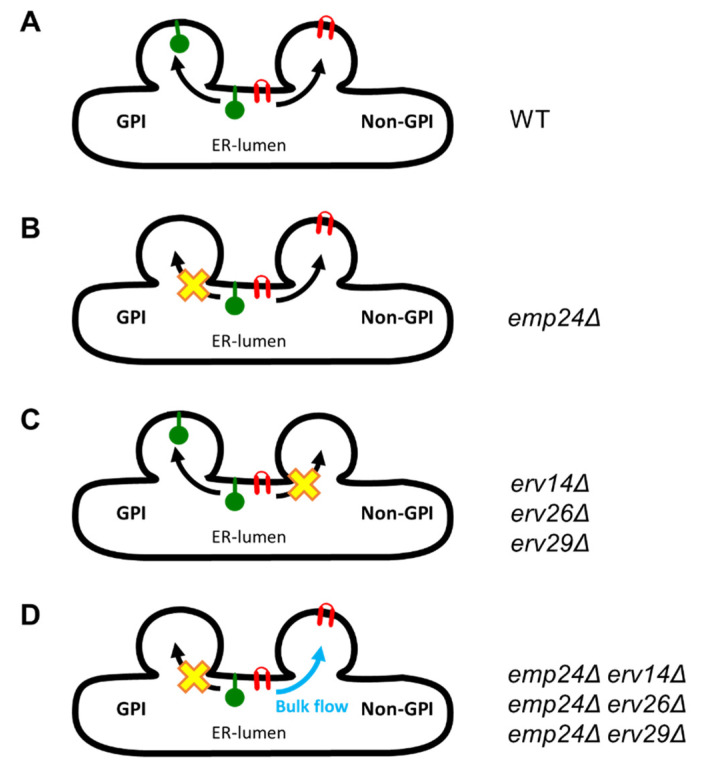
Model depicting the dual independent roles of the p24 complex in the selectivity of cargo export from the ER. (**A**) In wild-type cells, GPI-APs are exported from the ER via their specialized COPII-dependent pathway by the p24 complex and non-GPI-APs are transported by their respective cargo receptors through the general COPII-dependent pathway. (**B**) In the absence of the p24 complex (*emp24*Δ cells), GPI-AP export is selectively blocked at the ER whereas non-GPI-APs are still efficiently exported by their respective receptors. (**C**) Deletion of a non-GPI cargo receptor, like Erv14, Erv26 or Erv29, leads to the selective ER retention of its specific cargos. (**D**) When one of these non-GPI cargo receptors and Emp24 are simultaneously mutated (double mutant cells), GPI-AP pathway is blocked whereas the specific non-GPI cargos of the mutated receptor can now escape from the ER by bulk flow. This effect indicates that the p24 complex, in addition to acting as a specific cargo receptor in the GPI-AP export pathway, also functions by preventing the non-selective ER exit by bulk flow of non-GPI cargos.

This ability of the p24 complex to interact with other cargo receptors in a COPII dependent manner forming multireceptor complexes suggests that they can exit together the ER through the non-GPI-AP export pathway. This raises the question of the possible function of the p24 complex in this pathway. Our study reveals that the p24 complex might contribute to prevent the non-selective ER exit by bulk flow of non-GPI-AP secretory cargos (Figure 8). Indeed, we show that the p24 complex is required to retain in the ER different types of non-GPI-cargos when the corresponding receptor is missing. We also show that this secretory cargo retention role is independent from its role as cargo receptor for GPI-APs in their separated ER export pathway. In the non-GPI cargo receptor-p24 double mutant cells, the non-GPI cargo leaves the ER by bulk flow, whereas GPI-APs are still efficiently retained at the ER. Furthermore, we show that this non-selective cargo exit from the ER is not an indirect effect of the UPR, which is activated by the *EMP24* mutation. This is in agreement with previous observations that ER leakage of soluble chaperones in the absence of the p24 function occurred independently of the UPR [22,49]. Moreover, we have shown that an active Golgi-to-ER retrograde transport is not required for cargo retention at the ER in the receptor-p24 double mutant cells. Given that the p24 complex is involved in this retrieval pathway, it could be possible that in the absence of the p24 complex, ER escaped cargos via bulk flow could not be retrieved from the Golgi, and thus, progress through the secretory pathway. However, the impairment of the retrograde transport, produced by the thermosensitive allele of COPI *ret1-1*, could not release the cargo from the ER in the receptor mutant strains, indicating that the p24 complex contributes to the ER retention of cargos by preventing their export and not by recycling them from the Golgi. This is consistent with the previous observation that ER leakage of a soluble chaperone in the absence of the p24 function was observed in vitro using microsomes [14].

Previous studies have reported that the p24 complex contributes to the ER retention of resident proteins such as soluble chaperones and some misfolded non-GPI-APs [22,23]. The p24 complex has been postulated to act, in coordination with the COPII coat, as a selectivity filter by displacing the ER residents from COPII vesicles [22,55]. Furthermore, a recent study proposes that, since the p24 proteins are major COPII vesicle passengers with bulky luminal portions, their presence could sterically contribute to exclude ER residents from the constricted COPII vesicle [49]. In the present study, we show that, in addition to ER residents, also transmembrane and soluble secretory cargo proteins are prevented by the p24 complex to exit the ER via bulk flow. The postulated mechanism, by which the p24 complex creates luminal steric pressure, could also operate to exclude from the COPII vesicle those free, unbound secretory cargos that have not yet been actively captured by their corresponding receptor. We propose that the COPII-dependent formation of multireceptor complexes, in addition to promoting the collective sorting of non-p24 receptors with their bound-cargos as mentioned above, would also ensure the presence of the p24 complex into the same COPII vesicle to sterically preclude the non-selective incorporation of free, unbound cargos. Because the COPII machinery drives the formation of the multireceptor complexes, when a single receptor is mutated, the rest of receptors including the p24 complex still should oligomerize, and thus, prevent the bulk flow exit of the orphan cargos. Nevertheless, this and other possibilities, such as whether the COPII-dependent oligomerization of non-p24 receptors is influenced by the capture of their specific cargo, need to be experimentally addressed by future research.

Our observation that the secretory cargo protein Axl2 is functional and can be targeted to its correct destination when it escapes from the ER by bulk flow in the combined absence of the p24 complex and its cargo receptor Erv14 (Figure 5) suggests that the p24 complex might prevent the bulk flow ER exit of secretory cargo proteins in their native state. This possibility could have physiological implications. By obstructing the bulk flow exit of native and functional specific secretory cargos, the p24 complex may ensure their regulated transport through the mechanism of receptor-mediated capture. However, the rate of bulk flow of fluid and membrane has been reported to be high enough to support efficient transport through the secretory pathway [5,56,57]. This fact has raised the question of why receptor-mediated transport is favored over bulk flow for a subset of specific secretory cargos [5]. Several important physiological advantages have been proposed for active receptor-mediated cargo export from the ER, including the regulation of fine-tuning of protein deployment which should be important to couple secretory transport with signaling pathways, the increase of the transport rate for specific cargos that must rapidly reach their cellular destination or be rapidly removed from the ER, which is required for fast-growing cells, or to prevent the premature functional activation of the cargo which activity in the ER is detrimental for the cell (i.e., ligand binding or enzymatic activity) [5].

In conclusion, in our work, we describe that p24 complex associates in a COPII-dependent manner with other cargo receptors in multireceptor complexes. Furthermore, we show that the p24 complex contributes to the selectivity and fidelity of secretory cargo export from the ER through two independent mechanisms. In addition to acting as a specific cargo receptor in the specialized GPI-AP export pathway, the p24 complex also prevents the non-selective ER exit by bulk flow of transmembrane and soluble secretory cargos through the general ER export pathway. This last mechanism may favor native secretory cargo capture by receptors, which could count as an additional layer of regulation of selectivity during ER export.

## Figures and Tables

**Table 1:** *Saccharomyces cerevisiae* strains.

Strain	Genotype	Reference/Source
BY4742	*MATα his3 leu2 lys2 ura3*	Euroscarf
Y15936	*MATα erv29*Δ*::KanMx his3 leu2 lys2 ura3*	Euroscarf
Y14567	*MATα emp24*Δ*::KanMx his3 leu2 lys2 ura3*	Euroscarf
Y14421	*MATα erv14*Δ*::KanMx his3 leu2 lys2 ura3*	Euroscarf
Y16420	*MATα erv26*Δ*::KanMx his3 leu2 lys2 ura3*	Euroscarf
MMY679	*MATa emp24*Δ*::KanMx erv26*Δ*::KanMx his3 leu2 lys2 ura3*	This study
MMY680	*MATα emp24*Δ*::KanMx erv29*Δ*::KanMx his3 leu2 lys2 ura3*	This study
MMY1009	*MATα emp24*Δ*::hph erv14*Δ*::KanMx ade2 his3 leu2 lys2 trp1 ura3*	This study
MMY1271	*MATα ERV14-mCi::HIS5Sp sec16-2 ura3 leu2 his4 lys2 trp1*	This study
MMY1464	*MATα erv14*Δ*::KanMx ret1-1 his4 leu2 ura3*	This study
MMY1673	*MATα CPY-GFP::HISMx6 his3 leu2 lys2 ura3*	This study
MMY1675	*MATα emp24*Δ*::KanMx CPY-GFP::HISMx6 his3 leu2 lys2 met15 ura3*	This study
MMY1676	*MATα erv29*Δ*::KanMx CPY-GFP::HISMx6 his3 leu2 lys2 met15 ura3*	This study
MMY1679	*MATα emp24*Δ*::KanMx erv29*Δ*::KanMx CPY-GFP::HISMx6 his3 leu2 lys2 ura3*	This study
RH3042	*MATa ret1-1 his4 leu2 trp1 ura3*	H. Riezman
RH7016	*MATα ERV14-mCi::HIS5Sp his4 leu2 trp1 ura3*	H. Riezman
VGY448	*MATa EMP24-TAP::HIS3 his3 leu2 met15 ura3*	V. Goder

**Table 2:** Plasmids.

Plasmid	Description	Reference/Source
pJML01	*ori, CEN, ERV29-3xHA, LEU2*	C. Barlowe
pRH3083	*ori, CEN, CWP2-Venus, URA3*	H. Riezman
pRS416	*ori, CEN, GAS1-GFP, URA3*	L. Popolo
p3079	*ori, CEN, MID2-Venus, LEU2*	H. Riezman
pRS316	*ori, CEN, GNT1-GFP, URA3*	K. Kurokawa
p3055	*ori, CEN, GAP1-ub-GFP, URA3*	H. Riezman
pDN390	*ori, CEN, HAC1^i^, LEU2*	A. Spang

## Supplementary Materials

Supplementary materials can be found at https://www.mdpi.com/2073-4409/9/5/1295/s1, Figure S1: Mass spectrometry analysis of Emp24-TAP interactors, Figure S2: The p24 complex member Erv25 associates with the cargo receptor Erv14, Figure S3: The thermosensitive *ret1-1* mutation impairs the Golgi-ER retrograde transport at restrictive temperature, Table S1: Description of proteins identified by LCQ Deca XP Plus in Emp24-TAP samples by 2 or more peptides, Table S2: Peptides identified in Emp24-TAP samples analyzed by LCQ XP Plus analysis.

## Abbreviations

ER	endoplasmic reticulum
GPI-AP	Glycosylphosphatidylinositol-anchored protein
GFP	green fluorescent protein
mCi	m-citrine
UPR	unfolded protein response

## References

[1] Gomez-Navarro N., Miller E. (2016). Protein sorting at the ER-Golgi interface. J. Cell Biol..

[2] Dancourt J., Barlowe C. (2010). Protein sorting receptors in the early secretory pathway. Annu. Rev. Biochem..

[3] D’Arcangelo J.G., Stahmer K.R., Miller E.A. (2013). Vesicle-mediated export from the ER: COPII coat function and regulation. Biochim. Biophys. Acta.

[4] Geva Y., Schuldiner M. (2014). The back and forth of cargo exit from the endoplasmic reticulum. Curr. Biol..

[5] Barlowe C., Helenius A. (2016). Cargo Capture and Bulk Flow in the Early Secretory Pathway. Annu. Rev. Cell Dev. Biol..

[6] Marzioch M., Henthorn D.C., Herrmann J.M., Wilson R., Thomas D.Y., Bergeron J.J., Solari R.C., Rowley A. (1999). Erp1p and Erp2p, partners for Emp24p and Erv25p in a yeast p24 complex. Mol. Biol. Cell.

[7] Hirata R., Nihei C., Nakano A. (2013). Isoform-selective oligomer formation of Saccharomyces cerevisiae p24 family proteins. J. Biol. Chem..

[8] Nagae M., Hirata T., Morita-Matsumoto K., Theiler R., Fujita M., Kinoshita T., Yamaguchi Y. (2016). 3D Structure and Interaction of p24β and p24δ Golgi Dynamics Domains: Implication for p24 Complex Formation and Cargo Transport. J. Mol. Biol..

[9] Rojo M., Pepperkok R., Emery G., Kellner R., Stang E., Parton R.G., Gruenberg J. (1997). Involvement of the transmembrane protein p23 in biosynthetic protein transport. J. Cell Biol..

[10] Strating J.R., Martens G.J. (2009). The p24 family and selective transport processes at the ER-Golgi interface. Biol. Cell.

[11] Belden W.J., Barlowe C. (2001). Distinct roles for the cytoplasmic tail sequences of Emp24p and Erv25p in transport between the endoplasmic reticulum and Golgi complex. J. Biol. Chem..

[12] Aguilera-Romero A., Kaminska J., Spang A., Riezman H., Muñiz M. (2008). The yeast p24 complex is required for the formation of COPI retrograde transport vesicles from the Golgi apparatus. J. Cell Biol..

[13] Bremser M., Nickel W., Schweikert M., Ravazzola M., Amherdt M., Hughes C.A., Söllner T.H., Rothman J.E., Wieland F.T. (1999). Coupling of coat assembly and vesicle budding to packaging of putative cargo receptors. Cell.

[14] Castillon G.A., Aguilera-Romero A., Manzano-Lopez J., Epstein S., Kajiwara K., Funato K., Watanabe R., Riezman H., Muñiz M. (2011). The yeast p24 complex regulates GPI-anchored protein transport and quality control by monitoring anchor remodeling. Mol. Biol. Cell.

[15] Manzano-Lopez J., Perez-Linero A.M., Aguilera-Romero A., Martin M.E., Okano T., Silva D.V., Seeberger P.H., Riezman H., Funato K., Goder V. (2015). COPII coat composition is actively regulated by luminal cargo maturation. Curr. Biol..

[16] Muñiz M., Nuoffer C., Hauri H.P., Riezman H. (2000). The Emp24 complex recruits a specific cargo molecule into endoplasmic reticulum-derived vesicles. J. Cell Biol..

[17] Fujita M., Watanabe R., Jaensch N., Romanova-Michaelides M., Satoh T., Kato M., Riezman H., Yamaguchi Y., Maeda Y., Kinoshita T. (2011). Sorting of GPI-anchored proteins into ER exit sites by p24 proteins is dependent on remodeled GPI. J. Cell Biol..

[18] Bonnon C., Wendeler M.W., Paccaud J.P., Hauri H.P. (2010). Selective export of human GPI-anchored proteins from the endoplasmic reticulum. J. Cell Sci..

[19] Goder V., Melero A. (2011). Protein O-mannosyltransferases participate in ER protein quality control. J. Cell Sci..

[20] Sikorska N., Lemus L., Aguilera-Romero A., Manzano-Lopez J., Riezman H., Muñiz M., Goder V. (2016). Limited ER quality control for GPI-anchored proteins. J. Cell Biol..

[21] Satpute-Krishnan P., Ajinkya M., Bhat S., Itakura E., Hegde R.S., Lippincott-Schwartz J. (2014). ER stress-induced clearance of misfolded GPI-anchored proteins via the secretory pathway. Cell.

[22] Ma W., Goldberg E., Goldberg J. (2017). ER retention is imposed by COPII protein sorting and attenuated by 4-phenylbutyrate. Elife.

[23] Elrod-Erickson M.J., Kaiser C.A. (1996). Genes that control the fidelity of endoplasmic reticulum to Golgi transport identified as suppressors of vesicle budding mutations. Mol. Biol. Cell.

[24] Muñiz M., Zurzolo C. (2014). Sorting of GPI-anchored proteins from yeast to mammals--common pathways at different sites?. J. Cell Sci..

[25] Muñiz M., Riezman H. (2016). Trafficking of glycosylphosphatidylinositol anchored proteins from the endoplasmic reticulum to the cell surface. J. Lipid Res..

[26] Mayor S., Riezman H. (2004). Sorting GPI-anchored proteins. Nat. Rev. Mol. Cell Biol..

[27] Muñiz M., Morsomme P., Riezman H. (2001). Protein sorting upon exit from the endoplasmic reticulum. Cell.

[28] Castillon G.A., Watanabe R., Taylor M., Schwabe T.M., Riezman H. (2009). Concentration of GPI-anchored proteins upon ER exit in yeast. Traffic.

[29] Ballif B.A., Roux P.P., Gerber S.A., MacKeigan J.P., Blenis J., Gygi S.P. (2005). Quantitative phosphorylation profiling of the ERK/p90 ribosomal S6 kinase-signaling cassette and its targets, the tuberous sclerosis tumor suppressors. Proc. Natl. Acad. Sci. USA.

[30] Dieguez-Acuna F.J., Gerber S.A., Kodama S., Elias J.E., Beausoleil S.A., Faustman D., Gygi S.P. (2005). Characterization of mouse spleen cells by subtractive proteomics. Mol. Cell Proteom..

[31] Consortium U. (2019). UniProt: A worldwide hub of protein knowledge. Nucleic Acids Res..

[32] Rolli E., Ragni E., Calderon J., Porello S., Fascio U., Popolo L. (2009). Immobilization of the glycosylphosphatidylinositol-anchored Gas1 protein into the chitin ring and septum is required for proper morphogenesis in yeast. Mol. Biol. Cell.

[33] Belden W.J., Barlowe C. (1996). Erv25p, a component of COPII-coated vesicles, forms a complex with Emp24p that is required for efficient endoplasmic reticulum to Golgi transport. J. Biol. Chem..

[34] Herzig Y., Sharpe H.J., Elbaz Y., Munro S., Schuldiner M. (2012). A systematic approach to pair secretory cargo receptors with their cargo suggests a mechanism for cargo selection by Erv14. PLoS Biol..

[35] Bue C.A., Barlowe C. (2009). Molecular dissection of Erv26p identifies separable cargo binding and coat protein sorting activities. J. Biol. Chem..

[36] Margulis N.G., Wilson J.D., Bentivoglio C.M., Dhungel N., Gitler A.D., Barlowe C. (2016). Analysis of COPII Vesicles Indicates a Role for the Emp47-Ssp120 Complex in Transport of Cell Surface Glycoproteins. Traffic.

[37] Tanabe Y., Arai S., Wada I., Adachi H., Kamakura T., Yoda K., Noda Y. (2019). Svp26 facilitates ER exit of mannosyltransferases Mnt2 and Mnt3 in Saccharomyces cerevisiae. J. Gen. Appl. Microbiol..

[38] Shibuya A., Margulis N., Christiano R., Walther T.C., Barlowe C. (2015). The Erv41-Erv46 complex serves as a retrograde receptor to retrieve escaped ER proteins. J. Cell Biol..

[39] Noda Y., Hara T., Ishii M., Yoda K. (2014). Distinct adaptor proteins assist exit of Kre2-family proteins from the yeast ER. Biol. Open.

[40] Suda Y., Kurokawa K., Nakano A. (2017). Regulation of ER-Golgi Transport Dynamics by GTPases in Budding Yeast. Front. Cell Dev. Biol..

[41] Shindiapina P., Barlowe C. (2010). Requirements for transitional endoplasmic reticulum site structure and function in Saccharomyces cerevisiae. Mol. Biol. Cell.

[42] Kung L.F., Pagant S., Futai E., D’Arcangelo J.G., Buchanan R., Dittmar J.C., Reid R.J., Rothstein R., Hamamoto S., Snapp E.L. (2012). Sec24p and Sec16p cooperate to regulate the GTP cycle of the COPII coat. EMBO J..

[43] Yorimitsu T., Sato K. (2012). Insights into structural and regulatory roles of Sec16 in COPII vesicle formation at ER exit sites. Mol. Biol. Cell.

[44] Ishii M., Suda Y., Kurokawa K., Nakano A. (2016). COPI is essential for Golgi cisternal maturation and dynamics. J. Cell Sci..

[45] Roemer T., Madden K., Chang J., Snyder M. (1996). Selection of axial growth sites in yeast requires Axl2p, a novel plasma membrane glycoprotein. Genes Dev..

[46] Powers J., Barlowe C. (1998). Transport of axl2p depends on erv14p, an ER-vesicle protein related to the Drosophila cornichon gene product. J. Cell Biol..

[47] Belden W.J., Barlowe C. (2001). Deletion of yeast p24 genes activates the unfolded protein response. Mol. Biol. Cell.

[48] Ng D.T., Spear E.D., Walter P. (2000). The unfolded protein response regulates multiple aspects of secretory and membrane protein biogenesis and endoplasmic reticulum quality control. J. Cell Biol..

[49] Gomez-Navarro N., Melero A., Li X.H., Boulanger J., Kukulski W., Miller E.A. (2020). Cargo crowding contributes to sorting stringency in COPII vesicles. J. Cell Biol..

[50] Letourneur F., Gaynor E.C., Hennecke S., Démollière C., Duden R., Emr S.D., Riezman H., Cosson P. (1994). Coatomer is essential for retrieval of dilysine-tagged proteins to the endoplasmic reticulum. Cell.

[51] Mitrovic S., Ben-Tekaya H., Koegler E., Gruenberg J., Hauri H.P. (2008). The cargo receptors Surf4, endoplasmic reticulum-Golgi intermediate compartment (ERGIC)-53, and p25 are required to maintain the architecture of ERGIC and Golgi. Mol. Biol. Cell.

[52] Miller E.A., Beilharz T.H., Malkus P.N., Lee M.C., Hamamoto S., Orci L., Schekman R. (2003). Multiple cargo binding sites on the COPII subunit Sec24p ensure capture of diverse membrane proteins into transport vesicles. Cell.

[53] Bharucha N., Liu Y., Papanikou E., McMahon C., Esaki M., Jeffrey P.D., Hughson F.M., Glick B.S. (2013). Sec16 influences transitional ER sites by regulating rather than organizing COPII. Mol. Biol. Cell.

[54] Sato K., Nakano A. (2003). Oligomerization of a cargo receptor directs protein sorting into COPII-coated transport vesicles. Mol. Biol. Cell.

[55] Kaiser C. (2000). Thinking about p24 proteins and how transport vesicles select their cargo. Proc. Natl. Acad. Sci. USA.

[56] Thor F., Gautschi M., Geiger R., Helenius A. (2009). Bulk flow revisited: Transport of a soluble protein in the secretory pathway. Traffic.

[57] Fossati M., Colombo S.F., Borgese N. (2014). A positive signal prevents secretory membrane cargo from recycling between the Golgi and the ER. EMBO J..

