# Supplementary material for: Dual Independent Roles of the p24 Complex in Selectivity of Secretory Cargo Export from the Endoplasmic Reticulum
Source: Cells. 2020 May 22;9(5):1295. doi: 10.3390/cells9051295 (PMC7291304; doi:10.3390/cells9051295)
Supplement: Supplementary file 1 [file cells-09-01295-s001.zip › cells-741759 Supplementary data.pdf]

cells

MDPI

-Article

Dual independent roles of the p24 complex in selectivity of secretory cargo export from the endoplasmic reticulum

Sergio Lopez [1,3], Ana Maria Perez-Linero [1], Javier Manzano-López [1], Susana Sabido-Bozo [1,3], Alejandro Cortes-Garcia [1,3], Sofia Rodriguez-Gallardo [1,3], Auxiliadora Aguilera-Romero [1,3], Veit Goder [2] and Manuel Muñiz [1,3,*]

1 Department of Cell Biology, University of Seville, 41012 Seville, Spain
2 Department of Genetics, University of Seville, 41012 Seville, Spain
3 Instituto de Biomedicina de Sevilla (IBiS), Hospital Universitario Virgen del Rocío/CSIC/Universidad de Sevilla, 41012 Seville, Spain
* Correspondence: mmuniz@us.es

Supplementary data

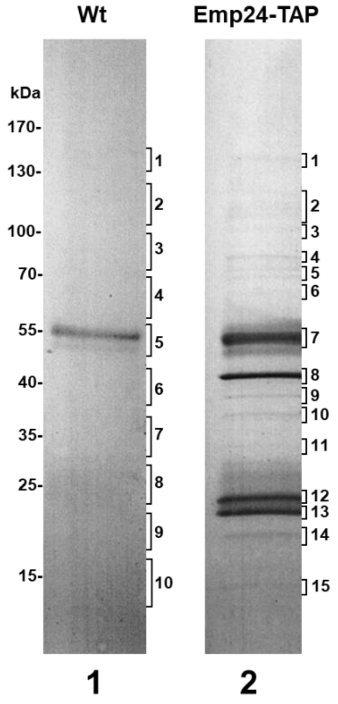

Figure S1. Mass spectrometry analysis of Emp24-TAP interactors. Enriched ER membrane fractions of wild-type yeast cells or cells expressing Emp24-TAP were isolated and solubilized with 1% digitonin. The extract was incubated with IgG-coupled magnetic beads and co-precipitated proteins were analyzed by SDS-PAGE and Coomassie Blue staining. Visualized bands (lane 2) and regions from the control reaction (lane 1) were numbered, cut out, subjected to in-gel digestion with trypsin and their protein content determined by LC-MS/MS techniques (Supplementary material Table S1, S2 and S3)

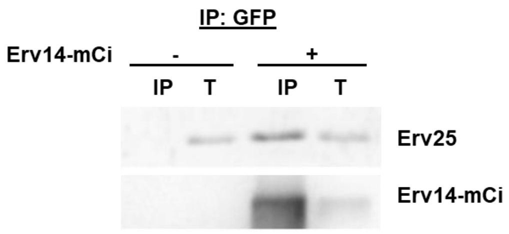

Figure S2. The p24 complex member Erv25 associates with the cargo receptor Erv14. Co-immunoprecipitation assay between Erv14-mCi and Erv25. Enriched ER membrane fractions of wild-type cells with or without expressing Erv14-mCi were solubilized, and immunoprecipitated (IP) with anti-GFP antibody, followed by immunoblotting with anti-Erv25 and anti-GFP antibodies. T represents a 0.5% of the solubilized input material.

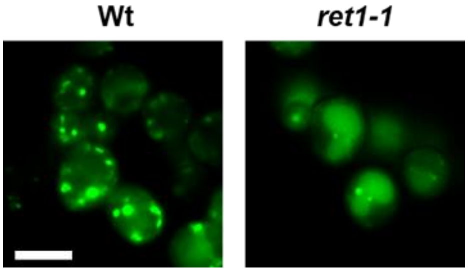

Figure S3. The thermosensitive *ret1-1* mutation impairs the Golgi-ER retrograde transport at restrictive temperature. Live images of wild-type and *ret1-1* cells expressing the recycling receptor Rer1-GFP at 37°C. In wild-type, Rer1-GFP showed the typical dotted Golgi pattern, whereas in *ret1-1*, it was mainly localized at the vacuole, indicating a defective retrograde transport. Scale bar, 5 μm.

Table S1. Description of proteins identified by LCQ Deca XP Plus in Emp24-TAP samples by 2 or more peptides,

Table S2. Peptides identified in emp24-TAP and control samples analyzed by LCQ XP Plus analysis.
